# Mental Health Across the Atrial Fibrillation Continuum: Mechanisms, Outcomes, and Clinical Implications

**DOI:** 10.3390/life16091399

**Published:** 2026-08-24

**Authors:** Aikaterini-Eleftheria Karanikola, Dimitrios Tsiachris, Georgia Balta, Panagiotis Alexandrou, Athanasios Dedousis, Fotis Tatakis, Michail Botis, Athanasios Kordalis, Konstantinos Tsioufis

**Affiliations:** First Department of Cardiology, National and Kapodistrian University of Athens, Hippokration General Hospital, 11527 Athens, Greece; elinakaranikola@gmail.com (A.-E.K.); georgiabalta@gmail.com (G.B.); alexandroupanos@gmail.com (P.A.); fotistatakis@yahoo.gr (F.T.); mgmpotis94@gmail.com (M.B.); akordalis@gmail.com (A.K.); ktsioufis@gmail.com (K.T.)

**Keywords:** atrial fibrillation, mental health, depression, anxiety

## Abstract

Atrial fibrillation (AF) is the most common supraventricular arrhythmia in adults, with a rising prevalence and a significant impact on quality of life and major cardiovascular outcomes. While atrial structural and electrical remodeling have long been central to AF pathophysiology, increasing evidence highlights the importance of psychological and mental health factors throughout the disease course. Depression, anxiety, and chronic stress are highly prevalent among individuals with AF and are associated with increased symptom burden, impaired quality of life (QoL), reduced treatment adherence, lower utilization of evidence-based therapies, and adverse clinical outcomes. Conversely, the diagnosis of AF itself may contribute to psychological distress, highlighting a complex bidirectional relationship between mental health and AF. This review summarizes current evidence on this interplay, with a focus on underlying mechanisms, including autonomic dysfunction and inflammation. Furthermore, the influence of mental health on outcomes and therapeutic strategies, and the clinical relevance of a multidisciplinary approach in contemporary AF management, are also examined.

## 1. Introduction

Atrial fibrillation (AF) is the most common sustained cardiac arrhythmia worldwide in the adult population. Its prevalence continues to rise owing to population aging, increasing survival from cardiovascular diseases, and the growing burden of cardiometabolic risk factors [1]. AF is associated with substantial morbidity and mortality, including an increased risk of stroke, heart failure, cognitive impairment, hospitalization, and mortality [2]. In addition to its impact on major cardiovascular outcomes, AF is frequently a highly symptomatic arrhythmia, causing palpitations, dyspnea, fatigue, dizziness, and exercise intolerance and resulting in a considerable reduction in quality of life and increased healthcare utilization [3].

Mental health disorders, including depression, anxiety disorders, bipolar disorder, schizophrenia, insomnia, and stress-related conditions, are highly prevalent and contribute substantially to the global burden of disease [4]. Beyond their effects on psychological well-being, increasing evidence suggests that mental health disorders exert important cardiovascular consequences through complex interactions involving autonomic dysfunction, inflammation, neurohormonal activation, behavioral factors, and healthcare utilization [5]. Consequently, mental health disorders are increasingly recognized as important determinants of cardiovascular health and disease.

The relationship between AF and mental health disorders has been increasingly recognized. Psychological distress has been associated with an increased risk of incident AF, while AF itself may lead to anxiety, depression, and impaired quality of life (QoL) [6]. Recent studies have reported prevalence rates of 24.3% for depression and 14.5% for anxiety among adults with AF, with substantially higher rates observed among women and older adults [7]. Furthermore, psychiatric comorbidities appear to influence symptom perception, treatment adherence, utilization and efficacy of rhythm-control and anticoagulation therapies, and long-term clinical outcomes [8]. These findings highlight the considerable psychological burden associated with AF and underscore the need for greater attention to mental health in this population. Beyond their effects on psychological well-being, depression, anxiety, and chronic stress have also been implicated as potential contributors to AF development and progression through shared pathophysiological mechanisms [8,9,10].

## 2. Objectives

This comprehensive review aims to summarize current evidence regarding the interplay between AF and mental health disorders, explore potential underlying mechanisms, examine their impact on treatment and clinical outcomes, and discuss therapeutic considerations and future directions for research and clinical practice.

## 3. Methods

The literature for this narrative review was identified through PubMed, Scopus, and Web of Science using Boolean operators and combinations of terms such as “atrial fibrillation”, “mental health”, “depression”, “anxiety”, “psychological distress”, “stress”, “quality of life”, “anticoagulation”, “catheter ablation”, “cognitive behavioral therapy”, “exercise”, and “lifestyle interventions”. Articles published in English between 2021 and 2026 were primarily considered, with older landmark studies included when considered relevant and highly contributory to the field. Both original research and review articles were screened to provide a comprehensive overview of current evidence. Priority was given to large observational studies, randomized controlled trials, systematic reviews and meta-analyses, and relevant consensus or guideline documents, particularly where these provided the strongest available evidence for clinical outcomes or therapeutic interventions. Titles and abstracts were independently screened by two authors, and citation tracking (snowballing) was used to identify additional relevant studies. All references were managed in a reference manager.

The review focuses on studies addressing the epidemiology, screening, pathophysiological mechanisms, clinical implications, and therapeutic considerations of the relationship between mental health disorders and AF. Given the broad and multidisciplinary nature of the topic, this work was conducted as a narrative rather than a systematic review; therefore, a PRISMA-based selection strategy was not applied. Due to this limitation, selection bias and incomplete literature inclusion cannot be fully excluded.

## 4. Assessment of Mental Health in AF Patients

Given the high prevalence of anxiety and depression among patients with AF and their negative effect on clinical outcomes, routine screening for psychological distress should be considered as part of comprehensive AF management. Early identification of mental health disorders may facilitate timely referral, initiation of appropriate treatment, and improved patient-centered care. This stepwise approach, followed by a referral to a mental health professional if positive, is also encouraged in the most recent ESC consensus on mental health and CVD [5].

Several screening instruments are available for use in clinical practice. Among the most widely used are the Patient Health Questionnaires (PHQ-2 and PHQ-9) and the Generalized Anxiety Disorder questionnaires (GAD-2 and GAD-7), which can be administered easily without licensing requirements [11]. The full 9-item and 7-item versions align with the DSM criteria for the respective disorders and have well-studied psychometric properties that render them trustworthy screening tools [12]. Although the shorter 2-item versions are less specific, they provide a time-efficient and sensitive preliminary screening step preceding the full versions, which can sometimes be time-consuming [13]. Similarly, the Whooley questions represent a simple two-item screening strategy that may serve as an initial assessment tool in busy clinical settings [14]. Another commonly used instrument is the Hospital Anxiety and Depression Scale (HADS), which includes separate seven-item subscales for anxiety and depression and has been extensively utilized in cardiovascular research [15]. Additional screening instruments are available, although many have been less extensively validated or may be less practical for routine clinical use (Table 1). Some tools have been specifically developed for older adults, such as the Geriatric Anxiety Inventory and Geriatric Depression Scale, which may be particularly relevant given the advanced age of many AF patients [16,17]. As for anger, there is less literature regarding screening tools and the most commonly used ones are the Spielberger Trait Anger Scale and the Dimensions of Anger Reactions Scale [18,19]. Similarly, for work stress, the Job Demand Control model has been used to assess mental strain and burnout in the workplace [20,21].

Overall, no single test has demonstrated clear superiority in patients with AF. However, given their extensive validation, ease of administration, and broad use in both clinical practice and research, the PHQ and GAD questionnaires may represent the most practical screening tools for depression and anxiety in this population.

Beyond psychological screening, assessment of overall well-being and QoL may provide complementary information. The World Health Organization Five Well-Being Index (WHO-5) is a brief instrument that evaluates subjective well-being and may also serve as a screening tool for depression [22]. In addition, AF-specific QoL instruments have been developed, including the Atrial Fibrillation Effect on QualiTy-of-Life (AFEQT) questionnaire and the Arrhythmia-Specific Questionnaire in Tachycardia and Arrhythmia (ASTA), both of which have demonstrated high detection rates of changes in AF-related health status and QoL [23].

**Table 1:** Screening tools for depression (D) and anxiety (A).

Screening Tool/Psychometric Property	Mental Health Condition	Sensitivity	Specificity	Item Number	Score Range (Cutoff)	Reference
Whooley questions	Depression	95%	65%	2	0–2 (≥1)	[24]
PHQ-2	Depression	97%	48%	2	0–6 (≥2)	[13]
PHQ-9	Depression	88%	88%	9	0–27 (≥10)	[25]
HADS-A	Anxiety	~89%	~75%	7	0–21 (≥8)	[26]
HADS-D	Depression	~80%	~88%	7	0–21 (≥8)	[26]
BDI-PC	Depression	83%	95%	7	0–21 (≥6)	[27]
BAI-PC	Anxiety	85%	81%	7	0–21 (≥5)	[27]
MDI	Depression	86–92%	82–86%	12	0–36 (≥26)	[28]
CESD	Depression	87%	70%	21	0–60 (≥16)	[29]
MHI-5	Mood and anxiety disorders	73–85%	73–83%	5	0–100 (≥76)	[30]
GAD-2	Anxiety	91%	37%	2	0–6 (≥3)	[13]
GAD-7	Anxiety	89%	82%	7	0–27 (≥8)	[31]
STAI-T	Anxiety	86%	57%	20	20–80 (≥44)	[32]
STAI-S	Anxiety	86%	53%	20	20–80 (≥40)	[32]
GAI	Anxiety	75%	84%	20	0–20 (≥11)	[17]

PHQ: Patient Health Questionnaire, HADS: Hospital Anxiety and Depression Scale, BDI-PC: Beck Depression Inventory for Primary Care, MDI: Major Depression Inventory, CESD: Center for Epidemiologic Studies Depression Scale, MHI-5: Mental Health Inventory-5, GAD: Generalized Anxiety Disorder questionnaire, STAI-T: State-Trait Anxiety Inventory-Trait, STAI-S: State-Trait Anxiety Inventory-State, GAI: Geriatric Anxiety Index.

## 5. Mental Health Disorders as Risk Factors for AF Development

### 5.1. Depression

Depression is one of the most extensively studied psychological risk factors for AF. Results from a recent meta-analysis of nine cohort and case–control studies suggest a 15% increase in AF risk among individuals with depression or depressive symptoms [33]. However, another meta-analysis of thirteen studies by Wu et al. reported an even higher risk of depression-associated incident AF, with an approximately 25% increase among participants [34]. Supporting these findings, the largest cohort study to date, conducted in a South Korean population, demonstrated a significantly higher cumulative incidence of AF among individuals with depression compared with healthy controls during a 10-year follow-up period (4.44% vs. 1.92%) [35]. Notably, the risk of new-onset AF was higher in patients with recurrent depressive episodes compared to those with single episodes (32% vs. 25%, respectively). The association was particularly pronounced among younger individuals and women, highlighting potential demographic differences in susceptibility [35].

### 5.2. Anxiety and Psychological Distress

Anxiety has also been implicated in AF development, although the available evidence is less consistent than that for depression. A recent meta-analysis reported a 10% increased risk of AF among individuals with anxiety disorders [34]. Anxiety has additionally been associated with higher rates of AF recurrence following catheter ablation in several studies [36,37]. Cheng et al. further demonstrated that panic disorder, a condition of extreme stress characterized by episodic autonomic activation, was independently associated with incident AF [38]. Chronic stress may also contribute to AF development, as a large prospective study of nearly one million veterans demonstrated that post-traumatic stress disorder (PTSD) was independently associated with a 13% increased risk of incident AF after adjustment for traditional cardiovascular risk factors and depression [39]. However, not all investigations have confirmed these findings, with several studies reporting no significant association between anxiety and AF risk [40,41]. These discrepancies may partly reflect differences in the definition and severity of anxiety, the use of symptom scales versus clinically diagnosed disorders, differences in study populations and follow-up duration, and variable adjustment for cardiovascular risk factors and behavioral confounders. Therefore, while anxiety may contribute to AF susceptibility based on some epidemiological evidence, the strength and consistency of this relationship remain uncertain and less clearly causal than that observed for depression.

Beyond anxiety disorders, other forms of psychological distress may also contribute to AF risk. In the Framingham Heart Study, psychological tension was associated with a 24% higher risk of incident AF in men, while anxiety was linked to increased all-cause mortality rather than AF development [42]. Similarly, occupational stress has been identified as a potential risk factor for AF, with evidence from prospective cohort studies and pooled analyses reporting an approximately 18–37% higher AF risk among individuals exposed to job strain [34,43]. Furthermore, anger and exposure to stressful situations have been examined as potential AF triggers. A retrospective study by Rosman et al., which included records from electronic devices from 2436 patients during the 2016 US presidential election, found an increase in several types of arrhythmias, including a 1.5-fold increase in AF risk [44]. Evidence from the Atherosclerosis Risk in Communities (ARIC) study suggests that psychological distress may be more strongly associated with AF risk than anger or social isolation, as individuals with high levels of vital exhaustion had a significantly increased risk of incident AF, whereas anger and poor social ties were not associated with AF occurrence [45]. On a more personal level, partner bereavement was associated with a transient increase in AF risk, particularly during the first weeks and up to one year following the loss and in cases of unexpected death in a large case–control study [46].

### 5.3. Other Mental Health Disorders

Emerging evidence suggests that the association between mental health disorders and AF may extend beyond depression, anxiety and stress-related conditions. Results from a nationwide cohort of more than 6.5 million young adults in Korea suggest that mental disorders were associated with a 53% higher risk of incident AF after multivariable adjustment. Notably, bipolar disorder and schizophrenia were associated with approximately a two-fold increase in AF risk, while depression, insomnia, and anxiety disorders conferred a 50–70% higher risk compared with individuals without mental illness [47]. However, data from a German registry yielded conflicting results, reporting a decreased incidence of AF among patients with schizophrenia [48]. In a recent bidirectional Mendelian randomization study, genetically predicted borderline personality disorder was associated with a modest but significant increase in AF risk, whereas no causal effect of AF on borderline personality disorder was observed, further supporting the hypothesis that certain psychiatric disorders may promote AF development [49].

Finally, sleep disturbances may also contribute to AF development. In a nationwide Japanese cohort including more than 1.7 million individuals, insomnia was associated with a 14% increased risk of incident AF after adjustment for potential confounders. Interestingly, the association was more pronounced in younger individuals and women, suggesting that insomnia may represent an underrecognized and potentially modifiable risk factor for AF [50]. Additional evidence comes from the Women’s Health Initiative study, which included more than 83,000 postmenopausal women and demonstrated that clusters of psychosocial stressors were independently associated with incident AF. Among individual factors, insomnia and stressful life events showed the strongest associations with AF risk, supporting a potential role for cumulative psychological stress in arrhythmogenesis [51]. Similarly, insomnia in the elderly has been linked with increased AF risk, as results from a cohort of 2428 patients showed [52].

Overall, the available evidence suggests that the association between mental health and AF extends beyond depression, encompassing anxiety disorders, psychological distress, bipolar disorder, schizophrenia, insomnia, and other psychiatric conditions. Nevertheless, the strength and consistency of the evidence vary considerably across disorders, with the most robust data currently available for depression (Table 2). Most available evidence linking mental health disorders with incident AF derives from observational cohort and case–control studies, which cannot establish causality. Consequently, the interpretation of these associations requires caution.

## 6. Pathophysiological Mechanisms Linking Mental Health Disorders and AF

The association between mental health disorders and AF is unlikely to be mediated by a single biological pathway. By contrast, evidence suggests that autonomic dysfunction, inflammation, neuroendocrine activation, oxidative stress, and even pharmacological factors interact to promote the structural and electrical remodeling that underlies AF.

One of the most extensively studied mechanisms is chronic low-grade inflammation and oxidative stress. Depression, anxiety, and chronic psychological stress have been associated with elevated circulating levels of pro-inflammatory cytokines, including tumor necrosis factor-α (TNF-α), interleukin-6 (IL-6), and C-reactive protein (CRP) [9,53]. Increased production of reactive oxygen species may induce mitochondrial dysfunction, cardiomyocyte apoptosis, and redox-mediated alterations in ion channel expression and function [54,55]. Ultimately, this pro-inflammatory state may lead to atrial remodeling and fibrosis, calcium handling abnormalities, cardiomyocyte apoptosis and electrophysiological instability, thereby creating an anatomical and electrophysiological substrate for AF development [56,57].

Another key component of the relationship between mental health disorders and AF is dysregulation of the autonomic and hypothalamic–pituitary–adrenal (HPA) systems. Psychological distress is associated with sympathetic hyperactivity and relative parasympathetic withdrawal, leading to catecholamine excess and adrenergic receptor overstimulation [10,58]. The resulting imbalance between the two systems promotes increased automaticity, higher rates of spontaneous depolarizations and non-uniform reduction in the effective refractory period, all of which contribute to arrhythmogenesis [59]. Neurohormonal activation through continuous stimulation of the HPA axis and sustained elevations of cortisol and other stress hormones may further augment these effects [60,61]. Persistent hypercortisolemia has been linked to systemic inflammation, oxidative stress, endothelial dysfunction, metabolic disturbances, and autonomic imbalance, all of which may contribute to atrial remodeling and arrhythmogenesis. Moreover, prolonged HPA-axis activation may interact with both the sympathetic nervous system and the renin–angiotensin–aldosterone system (RAAS), further promoting structural and electrical changes that facilitate AF development [61,62].

This neurohumoral-inflammatory response may also be amplified through the persistent overactivation of the RAAS that has been observed in patients with mental health disorders [63]. Increased angiotensin II signaling promotes fibroblast proliferation, reduces collagen degradation, and contributes to atrial fibrosis, chamber dilatation, and elevated atrial wall stress, thereby facilitating both structural and electrical remodeling [61]. At a molecular level, emerging evidence also suggests a role for dysregulated calcium/cyclic adenosine monophosphate (Ca^2+^/cAMP) signaling, which may contribute to both psychiatric disorders and AF through shared effects on intracellular signaling pathways and autonomic regulation [64].

In addition to these endogenous mechanisms, pharmacological treatment of mental health disorders may also promote arrhythmogenesis. Several psychotropic medications have been associated with adverse cardiovascular effects, including QT prolongation and autonomic dysregulation [65,66]. In particular, antipsychotic use has been linked to an increased risk of AF, potentially through interactions with multiple neurotransmitter receptors, particularly antagonism of cardiac M2 muscarinic receptors [66]. These interactions of biological consequences and pharmacotherapy highlight the complexity of the relationship between mental health and AF.

Taken together, all of the above findings support the hypothesis that chronic psychological stress and psychiatric disorders may influence AF susceptibility through interacting pathways. Importantly, however, most mechanistic evidence is derived from experimental studies, and direct evidence demonstrating that modification of these pathways prevents AF development in patients with mental health disorders remains largely underexplored. Therefore, even though these mechanisms provide some biological insights into the potential epidemiological associations, they should be interpreted with caution in respect to causality.

## 7. AF as a Risk Factor for Mental Health Disorders

While substantial evidence suggests that mental health disorders may contribute to AF development, the relationship appears to be bidirectional. AF is frequently associated with significant psychological distress, with patients experiencing higher rates of anxiety, depression, and impaired QoL than the general population [67].

Several studies have demonstrated a high prevalence of anxiety and depressive symptoms among patients with AF. A recent systematic review and meta-analysis of 26 studies reported pooled prevalence rates of 24.3% for depression and 14.5% for anxiety among adults with AF [7]. Supporting these results, a prospective study of 101 patients with AF suggested that approximately one-third exhibited clinically significant symptoms of depression and anxiety, with these symptoms persisting over a 6-month period. Importantly, depressive symptoms emerged as the strongest independent predictor of future QoL, highlighting the substantial psychological burden associated with AF [68]. Notably, the prevalence of depression and anxiety is higher among vulnerable demographic groups, including women and older adults, as results from a recent meta-analysis and several cohort studies suggest [7,69,70].

AF-related symptom burden appears to be a major contributor to psychological distress, with symptom clusters including palpitations, fatigue, dizziness, insomnia, and dyspnea being associated with higher levels of depression and anxiety in a cohort of 175 patients [71]. Emerging evidence suggests that the adverse impact of AF on QoL is influenced not only by symptom burden but also by illness perception, anxiety, and coping strategies, highlighting the importance of psychological adaptation to the disease [72]. Furthermore, AF symptoms may promote maladaptive coping behaviors, including exercise avoidance or kinesiophobia, through their effects on illness perception and psychological distress, thereby further impairing well-being [73].

Psychological distress is also common among patients with AF undergoing rhythm-control procedures. In a cohort study of 171 patients, moderate-to-severe depressive symptoms were reported in 17% of cases and clinically significant anxiety in approximately one-quarter to one-third of cases and were associated with greater AF symptom severity and poorer QoL [74]. Similarly, Kupper et al. studied 118 patients with persistent AF planned for electrical cardioversion and reported elevated levels of depression and anxiety among them, as assessed by BDI and STAI, both before and after the procedure [75].

The psychological burden of AF extends beyond arrhythmia symptoms. The diagnosis itself may be associated with fear and uncertainty, while concerns regarding stroke risk, bleeding complications, and the need for lifelong anticoagulation may further contribute to emotional distress [76]. In addition, the unpredictable nature of AF may lead to symptom preoccupation and heightened vigilance regarding cardiac symptoms. In a cohort of 409 patients with AF, symptom preoccupation was present in 37% of participants and was strongly associated with impaired QoL, greater symptom severity, and increased healthcare utilization [77].

Collectively, these findings suggest that anxiety and depression may not merely be comorbidities in patients with AF but may also be closely associated with the experience of living with a chronic condition. Symptom burden, uncertainty regarding disease progression, fear of complications, and treatment-related stress may contribute to psychological distress and poorer well-being, although the direction and mechanisms of these associations remain uncertain.

## 8. Impact of Mental Health on AF Outcomes and Prognosis

### 8.1. AF Prognosis

Beyond their potential role in AF development, mental health disorders may significantly affect disease progression, symptom burden, treatment outcomes, and long-term prognosis. More specifically, depression may negatively impact arrhythmia-free survival after catheter ablation, with a meta-analysis of seven studies concluding that patients with depression undergoing catheter ablation had more than twice the risk of AF recurrence compared with non-depressed individuals [78]. Additionally, although evidence regarding electrical cardioversion is more limited, available data similarly suggest an increased likelihood of AF recurrence among patients with depressive symptoms [79]. Beyond rhythm-control outcomes, depression has been associated with worse clinical outcomes in patients with AF. A large Japanese cohort study demonstrated that depression was independently associated with a 39% higher risk of ischemic stroke -particularly among younger patients-, even after adjustment for established cardiovascular risk factors and anticoagulant use [80]. Furthermore, depression has been associated with worse outcomes in patients with coexisting AF and heart failure, including increased cardiac mortality, arrhythmic death, and all-cause mortality [81]. Consistent with these findings, a large international registry demonstrated that symptoms of anxiety and/or depression were independently associated with higher risks of all-cause mortality, major adverse cardiovascular events, and cardiovascular death, with the risk increasing according to symptom severity [82]. Finally, depressive symptoms have been linked to greater AF symptom burden, with Rothe et al. reporting a three-fold increase in the likelihood of severe AF-related symptoms among patients with depressed mood at baseline [83].

Despite the aforementioned negative impact of psychological distress on AF outcomes, it appears that the benefit from rhythm-control interventions in these patients is retained. In a cohort of patients undergoing cryoballoon ablation, those with elevated anxiety or depressive symptoms reported lower QoL both before and after the procedure; however, improvements in QoL following ablation were comparable to those observed in patients without psychological distress [84].

### 8.2. Differences in Treatment Options

An important consideration in patients with mental health conditions is the potential disparities they face in receiving optimal treatment for various comorbidities, including AF. Data from a nationwide Finnish cohort (FinACAF) of more than 239,000 patients with incident AF showed that depression, anxiety disorders, bipolar disorder, and schizophrenia were all independently associated with lower use of rhythm-control strategies, including antiarrhythmic drugs, cardioversion, and catheter ablation [85]. The authors attribute these disparities to barriers to healthcare access, lower treatment adherence, difficulties in symptom assessment, and concerns regarding interactions between antiarrhythmic and psychotropic medications [85].

Additionally, mental health disorders may negatively affect the implementation of evidence-based stroke prevention strategies in AF. Large registry studies consistently tend to report lower rates of oral anticoagulant (OAC) initiation among patients with depression, anxiety disorders, bipolar disorder, schizophrenia, and other serious mental illnesses compared with those without psychiatric comorbidity [86,87,88]. This treatment gap appears particularly pronounced among patients with schizophrenia, severe psychiatric symptoms, substance use disorders, cognitive impairment, self-injurious behavior, and functional limitations [86,88]. Importantly, these disparities in OAC use have persisted despite the introduction of direct oral anticoagulants (DOACs) [86,87]. Beyond treatment initiation, maintaining effective anticoagulation may also present challenges in this population. A systematic review found that patients with serious mental illness were less likely to receive OAC therapy and, when treated with warfarin, often demonstrated poorer anticoagulation control [89].

The clinical relevance of these treatment disparities is highlighted by findings from the FinACAF registry, in which patients with mental health disorders exhibited higher crude rates of ischemic stroke. However, after adjustment for OAC use and other confounders, psychiatric comorbidity was no longer independently associated with incident stroke, indicating that differences in stroke prevention strategies may partly explain the excess risk observed in this population [90]. Several factors may contribute to the underutilization of anticoagulation, including concerns regarding medication adherence, cognitive impairment, and challenges associated with long-term treatment. Data regarding medication adherence in this subgroup are limited, even though some authors argue that compliance rates are comparable to those of the general population [91]. Furthermore, emerging evidence suggests that certain mental health disorders, particularly depression and anxiety disorders, are associated with an increased risk of bleeding events in patients with AF [92]. Collectively, these findings highlight the need for individualized assessment and equitable implementation of guideline-directed anticoagulation strategies in this vulnerable population.

## 9. Therapeutic Considerations and Clinical Implications

The coexistence of mental health disorders should not be regarded as a barrier to evidence-based AF treatment. By contrast, management should aim to address both psychiatric symptoms and AF using individualized, multidisciplinary strategies. For example, based on the shared pathophysiological mechanisms of depression and AF, effective treatment of depression could potentially influence AF management through modulation of autonomic function, improved adherence to lifestyle modifications, and reduced symptom perception [10]. Targeting depression and other mental health disorders with both pharmacological and behavioral interventions has shown favorable outcomes on psychological well-being and distress, supporting a more integrated approach to AF care [1,5].

### 9.1. Therapeutic Dilemmas and Considerations

Treatment of anxiety and depression in patients with AF should not be neglected because of concerns regarding potential cardiovascular effects. Although some observational studies have suggested an association between antidepressant use and incident AF, findings remain controversial, and it is often difficult to distinguish medication effects from the impact of the underlying psychiatric disorder itself [45,93,94]. Interestingly, a recent study reported that selective serotonin reuptake inhibitors and serotonin-noradrenaline reuptake inhibitors were associated with a lower AF burden among patients with paroxysmal AF, as detected by cardiac implantable electronic device data, raising the possibility that effective treatment of depression may favorably influence arrhythmia outcomes [95]. Consequently, current evidence does not support withholding appropriate antidepressant therapy solely because of concerns regarding AF risk. However, the potential drug–drug interactions and adverse effects associated with antidepressants, including a reported increased bleeding risk, should be considered in cases of concomitant use of antiarrhythmic or anticoagulant drugs and warrant close monitoring [96,97,98].

Emerging evidence suggests that successful rhythm-control strategies may offer important psychological benefits in addition to reducing arrhythmia burden. In the REMEDIAL randomized trial, catheter ablation was associated with significant reductions in anxiety, depression, and overall psychological distress compared with medical therapy alone, accompanied by a marked reduction in AF burden [99]. Similar findings have been reported in observational studies, where catheter ablation resulted in greater improvements in depression, anxiety, and QoL than antiarrhythmic drug therapy [100]. Furthermore, a large real-world cohort study demonstrated that AF ablation was associated with a reduced risk of developing subsequent psychiatric disorders, including anxiety, depression, insomnia, and suicidal ideation, compared with non-ablative management [101]. However, evidence regarding antiarrhythmic drug (AAD) use in patients with AF remains controversial, as many of the commonly used agents have been associated with neuropsychiatric effects. Case reports describe catatonic depression and psychosis-related symptoms linked to amiodarone initiation and discontinuation, particularly in older patients, even though the exact mechanism is unclear [102,103]. Additionally, medications used for rate control, including beta-blockers and calcium channel blockers, have also been historically associated with increased incidence of depression, anxiety, sleep disturbances and fatigue [104,105,106]. However, recent evidence argues against the neuropsychiatric effects of these drugs [107,108,109,110].

Another consideration in patients receiving AADs is the psychological burden that derives from the potentially life-threatening toxicities and significant side effects of these drugs. Patients with preexisting depression and anxiety should be monitored for symptom worsening when starting AADs, and drug interactions between AADs and psychotropic medications may further increase neuropsychiatric risk. For all of the above reasons, treatment decisions in patients with AF and psychiatric comorbidities should be approached in a multidisciplinary manner, incorporating both cardiovascular and mental health professionals.

### 9.2. Lifestyle Modification and Non-Pharmacological Interventions

Psychological interventions, particularly cognitive behavioral therapy (CBT), represent a promising adjunctive strategy for patients with AF and coexisting psychological distress. Several studies have demonstrated that CBT can improve AF-specific QoL, reduce anxiety, symptom preoccupation, avoidance behaviors, and illness-related distress, with benefits persisting for up to 12 months after treatment [111,112,113,114]. Notably, improvements in patient-reported outcomes often occur without significant changes in objective AF burden, suggesting that CBT may enhance patients’ ability to cope with symptoms and modify maladaptive cognitive and behavioral responses to the arrhythmia rather than directly affecting the arrhythmia itself [111,112]. Internet-delivered CBT has also been shown to have similar effects and additionally reduce healthcare utilization, highlighting its potential as an accessible and scalable intervention [111,112]. Furthermore, mindfulness-based and dyadic CBT approaches have been associated with improvements in patient satisfaction, psychological distress, and sense of coherence, supporting the broader role of psychosocial interventions in AF management [114]. Although the current evidence base remains relatively limited and consists largely of small studies, these findings support the integration of structured psychological interventions into comprehensive AF care, particularly for patients experiencing anxiety, depression, symptom preoccupation, or impaired QoL.

Beyond psychological interventions, lifestyle-based approaches may also provide mental health benefits in patients with AF. The role of exercise-based cardiac rehabilitation (ExCR) in improving QoL outcomes among patients with AF has been increasingly recognized. A recent meta-analysis including more than 2000 patients demonstrated that ExCR was associated with reduced AF recurrence, symptom burden, episode duration, and importantly, improvements in mental well-being, supporting the concept that comprehensive lifestyle interventions may simultaneously address both arrhythmia-related and psychological outcomes in these patients [115]. Specific exercise modalities, including yoga, may provide additional benefits. In a prospective study of patients with symptomatic paroxysmal AF, a 3-month yoga program was associated with reductions in AF burden, anxiety and depression scores, as well as significant improvements in multiple QoL domains, suggesting that mind–body interventions may represent a useful adjunct to conventional AF management [116]. However, physicians should consider the potential adverse effect that depression and anxiety may have on participation in lifestyle modification programs, thereby indirectly contributing to AF progression.

## 10. Gaps in Evidence and Future Directions

Although substantial progress has been made in understanding the relationship between mental health disorders and atrial fibrillation (AF), several important knowledge gaps remain. Most available evidence originates from observational studies and only a few randomized controlled trials. Mental health disorders frequently coexist with cardiovascular risk factors, socioeconomic disadvantage, and substance or alcohol abuse, making residual confounding difficult to exclude. Reverse causation is also possible, particularly for anxiety and depressive symptoms that may develop during the early stages of undiagnosed or paroxysmal AF. Future prospective studies are therefore needed to clarify the mechanistic pathways linking psychological distress and AF development and discover potential therapeutic targets. Mendelian randomization and other genetically oriented research studies could enhance understanding of the shared underlying pathophysiology of both diseases, but their findings remain dependent on the validity of genetic tools and assumptions regarding pleiotropy [117]. Furthermore, substantial heterogeneity exists in the definition and assessment of psychiatric conditions, ranging from clinical diagnoses and prescription records to self-reported symptoms and screening questionnaires. Consequently, the available evidence supports an association between several mental health conditions and AF, but the extent to which these disorders independently contribute to AF development remains incompletely established.

The identification of patients at risk represents another important area for future research. While several screening tools for depression and anxiety are available and easy to implement in clinical practice, evidence supporting routine mental health screening in AF populations remains limited. More evidence from randomized trials on whether systematic screening strategies improve diagnosis, QoL, adherence to therapy, disease progression and overall prognosis is needed.

In addition, the role of digital health technologies, wearable devices, and remote monitoring platforms in identifying psychological distress among patients with AF warrants further investigation. Wearables and smartphone-based ECG and mood-tracking tools offer an opportunity to capture real-time data linking psychological state to arrhythmia burden at home. Although they are increasingly used in patients with AF for rhythm monitoring, observational data suggest that they may be associated with greater symptom monitoring, increased healthcare utilization, and higher levels of anxiety and treatment-related concerns, underscoring the importance of careful patient counseling regarding their use [118]. Nevertheless, these devices may also offer important opportunities for patient-centered care through improved symptom tracking, increased disease awareness, earlier recognition of arrhythmia episodes, and more personalized management strategies, provided that appropriate education and clinical support are available. Future research should focus on improving this technology to explore whether acute stress, anxiety, or mood fluctuations are directly associated with AF episodes, and whether digital psychological interventions reduce symptomatic burden or healthcare contact.

Finally, recent interdisciplinary models have emphasized the need for a shift toward a more integrated, patient-centered approach to AF management. Collaboration between cardiologists, primary care physicians, psychologists, psychiatrists, and allied healthcare professionals may help address both the cardiovascular and psychological aspects of AF [119]. Such integrated care models may ultimately improve QoL, treatment adherence, and long-term outcomes in this growing patient population.

## 11. Conclusions

Mental health disorders and atrial fibrillation are closely connected through a complex and bidirectional relationship. Depression, anxiety, work-related stress, post-traumatic stress disorder, insomnia, and other forms of psychological distress have been associated with an increased risk of AF. At the same time, AF itself can contribute to the development of anxiety, depression, impaired QoL, and psychological distress. Shared mechanisms involving autonomic dysfunction, inflammation, neurohormonal activation, and behavioral factors have been implicated in these associations. Beyond their role in AF development, mental health disorders influence symptom burden, treatment utilization, patient satisfaction, and clinical outcomes. However, the extent to which psychiatric disorders independently contribute to AF development remains uncertain due to the largely observational nature of available evidence. Given the rising prevalence of both AF and mental health disorders worldwide, the need for large, prospective and randomized studies is highly relevant to determine whether systematic screening for psychological distress and targeted pharmacological and psychosocial interventions through a multidisciplinary approach can improve AF outcomes, QoL, and long-term prognosis.

## Figures and Tables

**Table 2:** Main evidence on the association between mental health disorders and AF development.

Mental Health Condition	Main Evidence	Evidence for Association with AF	Consistency	Key Clinical Implications	References
Depression	Multiple observational studies/meta-analyses	Moderate–strong	Relatively consistent	↑ AF incidence, symptom burden, recurrence and adverse outcomes	[33,34,35]
Anxiety/panic disorder	Observational studies/meta-analyses	Moderate	Some heterogeneity	↑ AF symptoms, QoL impairment and possibly AF incidence	[34,36,37,38,40,41]
Post traumatic stress disorder	Observational	Limited	Limited data	↑ AF risk	[39]
Anger/hostility	Observational	Limited	Inconsistent	Possible association with AF development	[44,45]
Work stress	Observational	Limited–moderate	Generally consistent	Possible increased AF incidence	[34,43]
Psychological distress	Observational	Limited	Limited data	Potential association with AF and psychological burden	[42,45,46]
Bipolar disorder/schizophrenia/borderline personality disorder	Cohort studies; Mendelian randomization studies	Limited	Conflicting	Potential association with AF	[47,48,49]
Sleep disturbances	Observational evidence	Limited	Relatively consistent	Potentially ↑ AF risk	[50,51,52]

## Data Availability

Not applicable.
